# Supplementary material for: Dynalign II: common secondary structure prediction for RNA homologs with domain insertions
Source: Nucleic Acids Res. 2014 Nov 21;42(22):13939–48. doi: 10.1093/nar/gku1172 (PMC4267632; doi:10.1093/nar/gku1172)
Supplement: SUPPLEMENTARY DATA [file supp_gku1172_nar-02021-z-2014-File012.zip › manual/Text/partition-cuda.html]

RNAstructure Command Line Help: partition-cuda


|  |  |  |
| --- | --- | --- |
|  | RNAstructure Command Line Help  partition-cuda | - Contents - Index |
| partition-cuda calculates a partition function for a sequence, which can be used to predict base pair probabilities. The code implements a subset of the functionality of partition. It is implemented using CUDA to run on graphical processing units (GPUs). USAGE: partition-cuda [options] <sequence, or file containing one> Required parameters: <seq file> \| The name of a sequence file containing input data or a sequence of composed of all A, C, G, U, and T (uppercase only). Note that lowercase nucleotides are NOT forced single-stranded in partition-cuda. Options which don't require added values: -d \| Specify that the sequence is DNA, and DNA parameters are to be used. Default is to use RNA parameters.; -h \| Display the usage details message.; -v \| Verbose, i.e. display the dynamic programming algorithm arrays for debugging. Options which require added values: -t \| Specify a file to which a matrix of base pairing probabilities will be written.; -l \| Specify a file to which -log10 base pairing probabilities will be written.; -p \| Specify a ct file name to which the ProbKnot structure will be written.; -m \| Specify the minimum helix length for a ProbKnot structure.; -b \| Specify a file name from which the thermodynamic parameters will be read. This file is generated by partition-save-params and is in binary format. This can save some startup time as compared to reading the ascii text parameter files, which is the default behavior. Workflow partition-cuda predicts base pairing probabilities. The -log10 base pairing probabilities data (written using-l) can be used to color-annotate predicted structures (see draw) or generate probaility plots (see ProbailityPlot). Alternatively, the program can predict ProbKnot structures (using -p), which can contain pseudoknots. Images can be made of structures (in ct format) using draw. If no file options are chosen, the program defaults to outputting the ct for the ProbKnot structure to standard out. The number of CUDA threads is set by a compile-time variable, NTHREAD. Two other compile-time variables THREAD\_X and THREAD\_Y set the dimensions of the block used for calculations of internal loops; the product of these must equal NTHREAD. By default, NTHREAD, THREAD\_X, and THREAD\_Y are set to 128, 8 and 16, respectively. References 1. Stern, H.A., and Mathews, D.H. "Accelerating calculations of RNA secondary structure partition functions using GPUs." *Algorithms Mol. Biol.*, 8:29. (2013). 2. Reuter, J.S., and Mathews, D.H. "RNAstructure: software for RNA secondary structure prediction and analysis." *BMC Bioinformatics*, 11:129. (2010). 3. Mathews, D.H. "Using an RNA secondary structure partition function to determine confidence in base pairs predicted by free energy minimization." *RNA*, 10:1178-1190. (2004). 4. McCaskill, J.S. "The equilibrium partition function and base pair probabilities for RNA secondary structure." *Biopolymers*, 29:1105-1119. (1990). | | |
| Visit The Mathews Lab RNAstructure Page for updates and latest information. | | |
